# East Suffolk and Ipswich Hospital

**Published:** 1924-01

**Authors:** 


### EAST SUFFOLK AND IPSWICH HOSPITAL.

### NURSES' PRIZE DAY.

THIS hospital, which has a staff of over 100 nurses, had its annual Nurses' Prize-giving Day on November 29. The distribution was made by Dame Maud McCarthy, G.B.E., R.R.C., Matron-in-Chief of the Territorial Army Nursing Service. The chair was taken by Mr. B. W. Elkington, the Chairman of the Board of Management of the hospital, supported by the Secretary, Mr. Arthur Griffiths, O.B.E., and several members of the Managing Committee and of the Visiting Staff.

### A Story of Rapid Growth.

The Chairman referred to the rapid growth of the hospital in recent years, and stated that when he joined the Board in 1907 the total amount of the salaries paid to the nursing staff amounted to £391 per annum, while the total expenditure of the hospital was less than £7,000 per annum. At the present time the salary bill for the nursing staff had increased to approximately £5000, while the annual revenue expenditure of the hospital averaged £40,000. Many of the nurses who had been through their training school had done uncommonly well in their subsequent professional career. He referred to the re-organisation of the syllabus of lectures by the various members of the Visiting Staff, and of the examinations which were now undertaken by Mr. Russell Howard, Dr. T. Grainger Stewart and Miss Ashdown, all of which tended to raise the status of the certificate issued by the hospital.

### The Qualified Nurse.

Referring to the Act for the State Registration of Nurses, he said that, although it was optional for any nurse to take or refuse such examination, yet if a nurse intended seriously to make nursing her life profession he strongly advised her to take it. At the present time there were in the country a great number of women wearing nurses' uniforms who were in no way qualified as nurses. This was manifestly unfair to those who had given up four of the best years of their life to arduous work and study in the hospitals in order to qualify for the profession. Whatever drawbacks there might be to State registration, it would undoubtedly do away with this anomaly. He regretted the unavoidable absence of Mr. John D. Cobbold, who had been Chairman of the Hospital for seven years, including the war period, and had done so much for the nursing service. Mr. Cobbold had given a hard tennis court for the use of the nurses. He had also given an endowment of £1,000 so that the income could be applied for the benefit of the nurses. Further, he had given the gold medals and other prizes for their examinations, and in many other ways he looked after the welfare of the nursing staff.

### A Happy Reunion.

The Examiners' reports were read and considered eminently satisfactory. All the nurses who had presented themselves for examination by Mr. Russell Howard, M.D., F.R.C.S., Lecturer on Surgical Nursing at the London Hospital, had passed; while of those who sat for the medical examination under Dr. Grainger Stewart, three had by a narrow margin failed to acquire the qualifying marks. The Matron, Miss K. V. S. Merriman, referred to her association with Dame Maud McCarthy five years ago on the battlefields of France. "We could always," she said, "turn for assistance—and never in vain—to our dear Matron-in-Chief, Dame Maud McCarthy."

### Wireless for Nurses.

The hospital has just completed its new Home for Nurses, which is delightfully situated in grounds adjoining the hospital, and provides a separate bedroom for each nurse with separate sitting-rooms comfortably furnished and supplied with a piano for each section of the nursing staff. A wireless installation has been added for the amusement of the nurses by the generous gift of Sir William Burton, one of the Vice-Presidents of the hospital. Arrangements have been made for training nurses from other hospitals, and an international scheme by which two Danish nurses are included in the staff has been adopted. If all the plans laid down for this branch of the hospital's work come to fruition there should be a great future for the nurses of the East Suffolk and Ipswich Hospital.

